# ACT2.6: Global Gene Coexpression Network in *Arabidopsis thaliana* Using WGCNA

**DOI:** 10.3390/genes16030258

**Published:** 2025-02-23

**Authors:** Vasileios L. Zogopoulos, Konstantinos Papadopoulos, Apostolos Malatras, Vassiliki A. Iconomidou, Ioannis Michalopoulos

**Affiliations:** 1 Center of Systems Biology, Biomedical Research Foundation, Academy of Athens, 11527 Athens, Greece; vzogopoulos@bioacademy.gr (V.L.Z.); kopap@biol.uoa.gr (K.P.); 2 Section of Cell Biology and Biophysics, Department of Biology, National and Kapodistrian University of Athens, 15701 Athens, Greece; veconom@biol.uoa.gr; 3 Molecular Medicine Research Center, biobank.cy, Center of Excellence in Biobanking and Biomedical Research, University of Cyprus, 2109 Nicosia, Cyprus; malatras.apostolos@ucy.ac.cy

**Keywords:** gene coexpression networks, WGCNA, *Arabidopsis thaliana*, microarrays, webtool

## Abstract

Background/Objectives: Genes with similar expression patterns across multiple samples are considered coexpressed, and they may participate in similar biological processes or pathways. Gene coexpression networks depict the degree of similarity between the expression profiles of all genes in a set of samples. Gene coexpression tools allow for the prediction of functional gene partners or the assignment of roles to genes of unknown function. Weighted Gene Correlation Network Analysis (WGCNA) is an R package that provides a multitude of functions for constructing and analyzing a weighted or unweighted gene coexpression network. Methods: Previously preprocessed, high-quality gene expression data of 3500 samples of Affymetrix microarray technology from various tissues of the *Arabidopsis thaliana* plant model species were used to construct a weighted gene coexpression network, using WGCNA. Results: The gene dendrogram was used as the basis for the creation of a new *Arabidopsis* coexpression tool (ACT) version (ACT2.6). The dendrogram contains 21,273 leaves, each one corresponding to a single gene. Genes that are clustered in the same clade are coexpressed. WGCNA grouped the genes into 27 functional modules, all of which were positively or negatively correlated with specific tissues. Discussion: Genes known to be involved in common metabolic pathways were discovered in the same module. By comparing the current ACT version with the previous one, it was shown that the new version outperforms the old one in discovering the functional connections between gene partners. ACT2.6 is a major upgrade over the previous version and a significant addition to the collection of public gene coexpression tools.

## 1. Introduction

Genes that exhibit similar expression patterns across multiple transcriptomic samples are considered as coexpressed [1]. Coexpressed genes tend to participate in similar biological processes, and gene coexpression patterns can provide insights into underlying cellular processes and can be used for the discovery of functional gene partners [2]. One of the most effective ways to study gene coexpression is known as ‘‘condition-independent’’ coexpression analysis, where the most representative transcriptomic samples of each tissue or cell type for a species of interest are selected [3], allowing for the study of global gene coexpression.

Networks represent interactions between nodes. Network-specific concepts, such as connectivity and modules, have proven valuable for the analysis of complex interactions [4]. The development of high-throughput technologies has allowed for network-based methods to be applied in many domains of biology. Gene coexpression is visualized through gene coexpression networks (GCNs), which are undirected graphs, depicting genes as nodes (vertices) and gene correlations as lines connecting gene pairs (edges) [5]. The Pearson correlation coefficient (PCC) is usually the metric of choice for measuring coexpression, and its thresholding results in unweighted networks known as “relevance networks” [4]. On the other hand, weighted networks are based on the strength of coexpression, through a “soft” thresholding. This involves defining adjacency functions to convert coexpression similarities into connection strengths and estimating parameters based on biological considerations [4]. Signed and unsigned networks may be produced, depending on whether the adjacency function retains the sign of gene correlations. By distinguishing between positive and negative correlations, signed networks provide a more nuanced view which can be valuable for understanding regulatory relationships and functional interactions in biological systems [6].

*A. thaliana* is a small, annual plant of the Brassicaceae family [7], valued as a model organism for genetic research. Its short life cycle, self-pollination ability, and small genome established it as a key subject for molecular studies [8]. *Arabidopsis* transitions from vegetative to reproductive growth [9], developing flowers through a complex process involving stem and floral tissue differentiation [10]. Its complete genome was sequenced in 2000 [7], aiding research on gene expression, development, and stress responses, with mutant screening [11] and transcriptome analyses [12], providing insights into phenotypic impacts and genetic functions. The influx of publicly available transcriptomic data has resulted in the development of many online resources for studying gene coexpression in *A. thaliana*. These include online gene coexpression tools and databases, which are often based on GCNs, such as ATTED-II [13], EXPath2.0 [14], ACT [15], AraNet [16], CORNET [17], GeneMANIA [18], ExpressionAngler [19], CoNekT [20], CoCoCoNet [21], etc. Furthermore, there are standalone pieces of software that enable GCN construction through the input of a user’s transcriptomic data, such as WGCNA [22], CoExpNetViz [23], scLink [24], LSTrAP [25], etc. These tools are not limited to a predetermined list of species and can be utilized to create *A. thaliana*-based GCNs using data originating from *A. thaliana* transcriptomic samples.

Weighted Gene Correlation Network Analysis (WGCNA) is a popular R package that contains methods for both weighted and unweighted gene coexpression network construction [26]. Weighted networks are preferred over unweighted networks, due to their capacity to preserve the intricate details present in the data, ensuring a more comprehensive representation of the relationships between genes [22]. Empirical and simulated studies have demonstrated the effectiveness of weighted network approaches in capturing meaningful biological relationships and producing more reliable results compared to unweighted networks, with WGNCA having numerous biological applications [27,28]. Moreover, WGCNA includes additional functionalities, such as grouping genes into modules and identifying intramodular hub genes. Consequently, a biological role may be attributed to a gene of unknown function based on the module to which it belongs [29], as gene members of a coexpression module may be involved in similar biological functions [22]. Finally, WGCNA is able to correlate gene expression patterns and sample traits [30].

This manuscript presents the new version (2.6) of the web-based *Arabidopsis* Coexpression Tool (ACT) which employs WGCNA to produce a microarray-based *A. thaliana* global coexpression landscape and discover gene coexpression modules. In the 2.6 version of ACT, the same gene expression data of the previous version (2.0) were re-analyzed through WGCNA, and ACT2.6 gene coexpression levels were calculated using the TOM-based similarity, instead of the Pearson correlation coefficient that was used in ACT2.0. The ACT2.6 web interface was updated to incorporate additional features which include gene coexpression module discovery, depiction of the module each gene belongs to, module-wide biological term enrichment analysis, association of modules with tissue traits, and identification of intramodular hub genes.

## 2. Materials and Methods

### 2.1. Data Acquisition

To study the global coexpression landscape in *A. thaliana*, a dataset of 3500 Affymetrix Arabidopsis ATH1 Genome Array chip microarray samples was used, as before [15,31]. In brief, samples analyzed with Affymetrix Arabidopsis ATH1 Genome Array chip were retrieved from the Gene Expression Omnibus (NCBI-GEO) [32], ArrayExpress (EMBL-EBI) [33], and NASCArrays [34] public repositories. Those samples were checked for possible duplicate or corrupted files, resulting in 19,887 unique samples. Raw “.cel” data were normalized via SCAN [35], a single-channel normalization algorithm, along with an updated BrainArray CDF file [36], with each sample including 21,273 probe sets that target unique genes. Samples that were retrieved from whole plants, retrieved from mutant plants, or low-quality were removed. Finally, the remaining samples were clustered according to the expression value of their genes, and using an iterative algorithm, similar samples were programmatically removed, down to having 3500 representative samples. This approach ensures a high signal-to-noise ratio within individual samples, decreased variation across samples, adjustment for batch and platform effects, and minimization of tissue bias.

The following biological terms were downloaded: gene descriptions from Thalemine [37], gene ontologies from Gene Ontology [38], plant ontologies from Planteome [39], biological and metabolical pathways from KEGG [40], AraCyc [41], and Wikipathways [42], transcription factor gene targets from AtRegNet [43] and Plant Cistrome Database [44], and protein domains from Pfam [45].

All downloaded and processed data, including normalized gene expression levels and metadata for each sample, were stored in a MySQL relational database.

### 2.2. WGCNA Analysis

To construct a weighted gene coexpression network, the WGCNA R package (version 1.71) was executed in the R console, running on a 14-core, 120 GB RAM, Linux Ubuntu 22.04 system. Gene expression data from the aforementioned 3500 representative microarray samples, as well as tissue metadata, were used as input for WGCNA. In order to be imported, tissue trait data for each sample were converted to a binary matrix. The WGCNA function pickSoftThreshold with parameter networkType = “signed” was used to estimate the value of soft-threshold power β as 14, which was used to calculate adjacency values and, subsequently, Topological Overlap Matrix (TOM) similarity and distances between all genes. Average linkage hierarchical clustering was used to create a gene dendrogram that was exported in Newick format [46]. Genes were grouped into modules with dynamicTreeCut [47] based on the coexpression gene dendrogram, with parameters minClusterSize set as 15 and deepSplit as 2. Modules were grouped into a dendrogram using average linkage, and modules with a height <0.25 were merged. The merged modules, represented as eigengenes, were associated with tissue traits by calculating PCCs between the eigengenes and samples from the same tissue type (trait).

### 2.3. Website Construction

The web server is hosted on a Linux Ubuntu 22.04, 16-core, 64 GB memory system and served through Apache2. The web application was developed using HTML5, CSS, Bootstrap 5, PHP, and Javascript. All programming scripts were developed in PHP.

Initially, the user selects an *A. thaliana* gene, called the “driver” gene, through an auto-completed field, based on the genes included in the webtool database. ACT2.6 outputs a gene coexpression clade which includes the driver gene and its coexpressed gene partners. The default clade size is defined using an iterative algorithm, pruning internal nodes until the coexpression clade is the closest to containing 25 genes. Trial and error showed that this choice of the number of genes is optimal. Nevertheless, the clade size may be modified by adding internal nodes, up to a maximum of 25% of the total genes, or removing them, down to a single clade. At the top of the clade depiction, a scale bar, which corresponds to TOM-based distances between genes, is displayed. The driver gene is highlighted in yellow. The user may change the driver gene by clicking on a different AGI code, while clicking on the gene symbol redirects to the gene’s page in Thalemine.

The webtool allows users to perform relevant gene term overrepresentation analyses by selecting an enrichment analysis type from a drop-down menu. These analyses are executed using as input the genes which are included in the currently selected subtree. The results, including overrepresented biological terms such as gene or plant ontologies, pathways, gene-targeting transcription factors, and protein domains, are summarized in a term enrichment table. *p*-values are calculated based on the Hypergeometric Distribution [48], and the terms are ranked according to their False Discovery Rate (FDR)-adjusted *p*-values [49]. Only terms with an FDR-adjusted *p*-value ≤ 0.05 are displayed.

For each enriched term, the analysis presents the hit percentage (the frequency of the term’s occurrence in the subtree compared to its overall occurrence in the dataset) and the overrepresentation rate (observed versus expected frequency). The size of the subtree influences the results, as increasing its size may reveal additional enriched terms not detected in smaller subtrees. A larger subtree may encompass gene subclades with diverse functions, whereas smaller subtrees yield more specialized enrichment results. Monitoring the variation in enrichment *p*-values can help identify the optimal subtree size for analysis.

A second table provides a comprehensive list of the genes in the subtree along with all associated terms within the selected category, with hyperlinks to their source databases. The gene list for the subtree can be downloaded for use in external tools such as WebGestalt [50] or redirected for further analysis via links to platforms like STRING [51], Thalemine, g:Profiler [52], and Flame [53].

### 2.4. New Features in ACT2.6

In ACT2.6, the module each gene belongs to is now displayed as a colored circle next to the gene name in the gene coexpression clade. By clicking on the gene module circle, the user is redirected to the corresponding module page. On that page, the statistically significant positive or negative correlations of the module’s eigengene with each tissue trait are displayed. In addition, the enriched biological terms of that module’s genes for each biological term category are shown. Finally, a table at the bottom of the page shows all the genes of the module, ranked by their “average ranking”. The average ranking of gene *i* $\left({avgRank}_{i}\right)$ in a module is calculated as follows:

$${avgRank}_{i}=\frac{\left(n+1\right)}{\left(n-1\right)\left(n\left(n-1\right)/2+1\right)}\sum_{j=1}^{n}R_{i,j}$$

where *i* and *j* are genes of the module, *n* is the number of genes in the module, and $R_{i,j}$ is the rank of the distance between genes *i* and *j* in the list of distances between all $n\left(n-1\right)/2$ pairs of the genes of the same module. In each module, the top-ranking gene and the genes having an avgRank difference from that of the top-ranking gene <1 are considered as that module’s intramodular hub genes.

### 2.5. API Access

ACT2.6 offers public access to coexpression clades and enrichment analyses via a JSON-based Application Programming Interface (API). The API is keyed on an *A. thaliana* AGI code, a tree node number, and optionally, a two-character keyword representing an enrichment analysis category. For instance, the URL https://www.michalopoulos.net/act2.6/api/AT3G16920/5/bp (accessed on 8 January 2025) retrieves the coexpression clade in Newick format for AT3G16920 as the driver gene with 5 internal nodes, details about the driver gene, a list of genes within the coexpression clade, and enriched “Gene Ontology: Biological Process” terms ranked by adjusted *p*-value. If an incorrect or missing keyword is provided, the enrichment analysis will not be executed. Detailed instructions for API usage are available in the Help section of the ACT2.6 website.

### 2.6. Comparison Between the Current and Previous ACT Versions

ACT2.6 and ACT2.0 were benchmarked with the same 10 gene use cases (*AT4G13170*, *HSP101*, *COR15A*, *CEV1*, *CTL2*, *PSB28*, *LHY*, *PSBT*, *AMS*, and *emb1692*) that were originally presented in ACT2.0 [15]. For comparison impartiality, the same biological term database versions were used, while the number of coexpressed genes was kept as close as possible, since an identical number of resulting coexpressed genes between the two versions cannot always be achieved, due to the coexpression tree representation being pruned based on internal node number.

## 3. Results

### 3.1. Gene Module Generation

**Figure 1:**
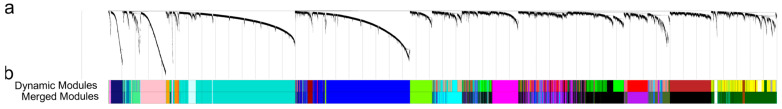
WGCNA-constructed gene coexpression tree and generated modules: (**a**) Gene coexpression tree created using the TOM-based distances through average linkage. Each leaf corresponds to a different gene. (**b**) Original 42 modules produced by dynamicTreeCut (top row) and 28 modules produced after the merging of the original modules (bottom row). Each module is depicted with a different color.

The resulting gene coexpression dendrogram contained 21,273 *A. thaliana* genes, originally grouped into 42 gene modules. After module merging, 28 modules (27 functional modules, with gene numbers ranging from 27 to 3809, and 1 module that contained 2 ungrouped genes) were generated (Figure 1).

**Figure 2:**
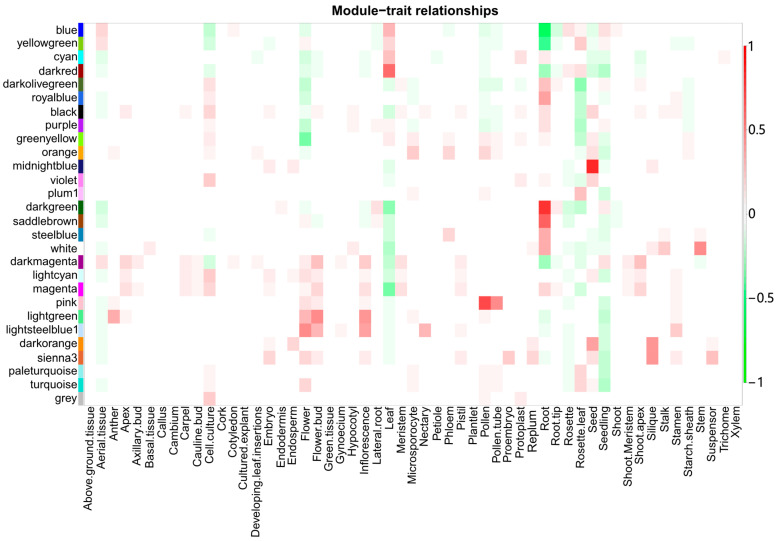
Heatmap depicting the Pearson correlation coefficient-based associations between merged gene modules and tissue traits.

The PCCs between the eigengenes describing each of the 28 merged modules and the 55 tissue traits were calculated (Figure 2), allowing for the overall expression pattern of the genes of each module to be associated with specific healthy tissues/plant parts.

### 3.2. Functional Exploration of ACT2.6 WGCNA Modules

The enrichment analysis of a series of general or plant-specific biological terms, using ACT2.6’s internal enrichment functionality, was performed for all 27 functional gene modules, with GOBP enrichment being mainly used to indicate each module’s predominant biological function. In 14 out of 27 modules, there is an accordance between the top GOBP term and the GOBP terms that characterize the hub gene, and in 17 out of 27 modules, there is bibliographic evidence for the overexpression of the hub gene, in the module’s overexpressed tissue (Table 1). All modules are described [54] (pp. 13–60). Six indicative modules of variable sizes are also described in this manuscript.

#### 3.2.1. Lightsteelblue1 Module

The lightsteelblue1 module includes 27 genes. They are overexpressed in “Flower”, “Inflorescence”, “Nectary”, “Flower bud”, “Stamen”, “Pistil”, and “Gynoecium” tissues and underexpressed in “Seedling” and “Root” tissues. All of these genes are associated with flower and/or anthesis. “Stamen filament development”, “jasmonic acid mediated signaling pathway”, “terpene biosynthetic process” and “nectar secretion” GO terms are significantly overrepresented (Table 2).

*MYB21* (myb domain protein 21) and *AT5G44630* (Terpenoid cyclases/Protein prenyltransferases superfamily protein) showed up as intramodular hub genes. *MYB21* and *MYB24* (myb domain protein 24), two transcription factors belonging to the lightsteelblue1 module, are the only known regulators of jasmonate, which is necessary for the development of stamen and pollen in *Arabidopsis* [55]. A *MYB21* mutant plant showed reduced male fertility, delayed anther dehiscence, and shorter anther filaments. Although the *MYB24* mutant plant appeared normal, the double *MYB21/MYB24* mutant showed severe defects in all three aspects of stamen development. Exogenous jasmonate was ineffective at restoring male fertility in either the *MYB21* or *MYB21*/*MYB24* mutant plants. *MYB21* and *MYB24* are induced by jasmonate and play a crucial role in regulating various aspects of stamen development in *Arabidopsis* [55].

*AT5G44630*, the other hub gene of this module, is one of the main genes responsible for the production of sesquiterpenes that are emitted from *Arabidopsis* flowers [73]. TPS14 (terpene synthase 14) and AT3G25810 (Terpenoid cyclases/Protein prenyltransferases superfamily protein) are terpene synthases that are responsible for the synthesis of alcohol linalool and monoterpene volatile products, respectively, in flowers (Table 2) [73]. Another two genes of the module, *CYP76C3* (cytochrome P450, family 76, subfamily C, polypeptide 3) and *CYP71B31* (cytochrome P450, family 71, subfamily B, polypeptide 31), code for P450 cytochrome enzymes which metabolize the two linalool enantiomers to form hydroxylated or epoxidized compounds [74]. *CYP76C3* and *CYP71B31* are shown to be coexpressed with *TPS10* (terpene synthase 10) and *TPS14* [74], with the latter two being terpene synthases of this module.

**Figure 3:**
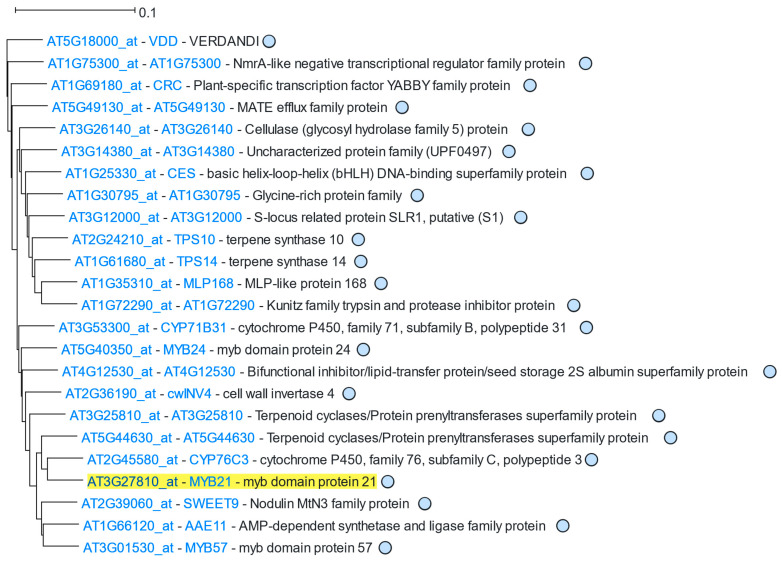
ACT2.6 *MYB21* 11 internal node coexpression clade. The driver gene (*MYB21*) is highlighted in yellow.

*MYB21* was used as input for ACT2.6, and the produced coexpression clade was set to 13 internal nodes, containing 24 genes (Figure 3). All genes of this clade belong to the lightsteelblue1 module, including the two hub genes of this module (*MYB21* and *AT5G44630*), which are located close to each other. GO enrichment analysis revealed enriched terms related to terpene biosynthesis, in accordance with the enriched terms of lightsteelblue1 module (Table 2), as 24 out of the 27 genes of this module are part of this clade.

#### 3.2.2. Yellowgreen Module

The yellowgreen module includes 45 genes, all of which are found in the same clade of the gene coexpression tree. These genes are exclusively located on the chloroplast chromosome. This module is overexpressed in “Rosette leaf”, “Leaf”, “Aerial tissue”, and “Seedling” tissues and underexpressed in “Root”, “Cell culture”, “Root tip”, “Seed”, “Starch sheath”, “Pollen”, and “Lateral root” tissues. The enrichment analysis of this module indicated the prevalence of specific biological process terms, such as “photosynthesis” and “electron transport chain”, which were significantly overrepresented (Table 3).

Hub genes *YCF4* and *ATPF* are the only plastid-related genes that are overexpressed in multiple cases of transgenic plants that overexpress *RAP2.2* [75], although the latter belongs to another module (purple).

Chloroplasts are essential for photosynthesis. As cell organelles, they feature their own chromosome, and their genes are organized as operons or transcriptional units [56]. *ATPA* (ATP synthase subunit α), *ATPI* (ATPase, F0 complex, subunit A protein), and hub genes *ATPH* (ATP synthase subunit C family protein) and *ATPF* (ATPase, F0 complex, subunit B/B′, bacterial/chloroplast) are organized into the ATP synthase (*atp*) operon. *ATPH* transcripts are more abundant compared to all other *atp* operon transcripts because of the protection of their structure, as they possess both a hairpin structure at the 3′ end and RNA-binding proteins at the 3′ end and at the 5′ end [56].

*PSBA* (photosystem II reaction center protein A), *PSBB* (photosystem II reaction center protein B), *PSBC* (photosystem II reaction center protein C), and *PSBD* (photosystem II reaction center protein D), which code proteins of photosystem II protein complex [76], belong to this module, resulting in the enrichment of “photosystem II protein” Pfam domain (Table 3). PSBC and PSBD are encoded by the same polycistronic transcript [76].

#### 3.2.3. White Module

The white module contains 102 genes, 99 of which are located in the same clade. They are overexpressed in “Stem”, “Root”, “Stalk”, “Hypocotyl”, “Basal tissue”, “Silique”, and “Replum” tissues and underexpressed in “Leaf”, “Rosette leaf”, “Seed”, “Seedling”, “Aerial tissue”, and “Rosette” tissues. The “plant-type secondary cell wall biogenesis” and “xylan metabolic process” GO terms and “xylem” PO term are significantly overrepresented (Table 4). *IRX3* (cellulose synthase family protein) and *GAUT12* (galacturonosyltransferase 12) are the two hub genes of this module.

Many genes contained in the white module, such as *IRX3*, *IRX12* (Laccase/Diphenol oxidase family protein), *IRX9* (Nucleotide-diphospho-sugar transferases superfamily protein), *CESA4* (cellulose synthase A4), *IRX6* (COBRA-like extracellular glycosyl-phosphatidyl inositol-anchored protein family), *GXM3* (glucuronoxylan 4-O-methyltransferase-like protein, DUF579), *CTL2* (chitinase-like protein), *KNAT7* (homeobox knotted-like protein), *PGSIP3* (plant glycogenin-like starch initiation protein 3), *GAUT12, AT4G27435* (fiber, DUF1218), *GUT2* (Exostosin family protein), *IRX1* (cellulose synthase family protein), *AT5G60720* (electron transporter, putative (Protein of unknown function, DUF547), *AT1G72220* (RING/U-box superfamily protein), *RWA1* (O-acetyltransferase family protein), *RIC2* (ROP-interactive CRIB motif-containing protein 2), *AT1G08340* (Rho GTPase activating protein with PAK-box/P21-Rho-binding domain-containing protein), *PGSIP1* (plant glycogenin-like starch initiation protein 1), *FLA11* (FASCICLIN-like arabinogalactan-protein 11), and *LAC2* (laccase 2) were known to exhibit similar expression patterns [63].

*IRX3* is highly expressed in the hypocotyl and in the base of the stem, while in samples from bigger height points of the stem, it shows lower expression levels [63]. In addition, *ERF38*, which belongs to a different module (blue), has been shown to be coexpressed with a series of white module genes, i.e., *AT1G07120* (CHUP1-like protein), *AT1G09440* (Protein kinase superfamily protein), *AT1G22480* (Cupredoxin superfamily protein), *RIC2, GUT2, ATMYB103* (myb domain protein 103), *AT1G80170* (Pectin lyase-like superfamily protein), *LAC2, AT2G31930* (hypothetical protein), *AT2G40120* (Protein kinase superfamily protein), *AT2G41610* (transmembrane protein), *IQD10* (IQ-domain 10), *CTL2, PGSIP1, IRX15* (IRREGULAR XYLEM protein (DUF579)), *AT3G59845* (Zinc-binding dehydrogenase family protein), *NAC073* (NAC domain-containing protein 73), *FLA11, AT5G06930* (nucleolar-like protein), *IRX6, CESA4*, and *FLA12* (FASCICLIN-like arabinogalactan-protein 12), which are related to secondary cell wall processing. The expression of *ERF38* at floral stems and mature siliques is intensive and may participate in an alternative, non-typical with cellulose and lignin, process of secondary cell wall synthesis [77], possibly explaining its grouping in a different module.

#### 3.2.4. Midnightblue Module

The midnightblue module contains 385 genes, 374 of which are located in the same clade. They are overexpressed in “Seed”, “Endosperm”, “Silique”, and “Embryo” tissues and underexpressed in “Leaf”, “Seedling”, and “Rosette” tissues. “Seed development”, “seed maturation”, and “seed oilbody biogenesis” are overrepresented GOBP terms (Table 5).

*AT1G27990* (transmembrane protein) and *AT1G72100* (late embryogenesis abundant domain-containing protein/LEA domain-containing protein) are the hub genes of this module. Late embryogenesis abundant (LEA) genes activate during various stresses and accumulate at late stages of seed development [67]. Tandem repeat genes *AT3G22490* and *ATECP31* (*AT3G22500*, LATE EMBRYOGENESIS ABUNDANT PROTEIN ECP31), which encode seed maturation proteins, have a positive correlation of their expression levels [78].

Gene pairs *AtLEA4-1* (Late Embryogenesis Abundant 4-1) and *LEA18* (Late Embryogenesis Abundant 18), *LEA7* (LATE EMBRYOGENESIS ABUNDANT 7) and *AT3G15670* (late embryogenesis abundant protein), *AT1G72100* and *AT1G22600* (late embryogenesis abundant protein), *AT2G18340* (late embryogenesis abundant domain-containing protein) and *AT4G36600* (late embryogenesis abundant protein), *LEA* (dehydrin LEA) and *AT4G39130* (Dehydrin family protein), *ECP63* (embryonic cell protein 63) and *AT3G53040* (late embryogenesis abundant protein), and *AT4G21020* (late embryogenesis abundant protein) and *AT5G44310* (late embryogenesis abundant protein) were found to have similar expression patterns, with all of them being expressed in seeds [78], serving as an additional line of evidence for the grouping of those genes in the same module.

Multiple genes of the midnightblue module reach their maximum expression in the mid to late seed developmental stages, albeit having different points of initial expression [79]. *AT1G03890* (RmlC-like cupins superfamily protein), *PER1* (1-cysteine peroxiredoxin 1), *AT1G65090* (nucleolin), *LBD40* (LOB domain-containing protein 40), *TIP3;1* (Aquaporin-like superfamily protein), *CBSX4* (Cystathionine β-synthase family protein), *AT3G01570* (oleosin family protein), *OLEO4* (oleosin 4), *AT3G63040* (hypothetical protein), *OLEO1* (oleosin 1), *SESA2* (seed storage albumin 2), *ATS3* (embryo-specific protein 3), *OLEO2* (oleosin 2), *SESA5* (seed storage albumin 5), *ATPXG2* (peroxygenase 2), *CYP71B10* (cytochrome P450, family 71, subfamily B, polypeptide 10), *AT3G54940* (Papain family cysteine protease), *AT5G59170* (Proline-rich extensin-like family protein), and *AT5G62800* (protein with RING/U-box and TRAF-like domain) start being expressed during the early stages of seed development (embryos at early heart to late torpedo stage). *SOM* (Zinc finger C-x8-C-x5-C-x3-H type family protein), *AT1G14950* (Polyketide cyclase/dehydrase and lipid transport superfamily protein), *AT1G48660* (Auxin-responsive GH3 family protein), *GLYI8* (Lactoylglutathione lyase/glyoxalase I family protein), *AT2G33520* (cysteine-rich/transmembrane domain protein A), *SMP1* (seed maturation protein 1), *XTH11* (xyloglucan endotransglucosylase/hydrolase 11), *CYP76C7* (cytochrome P450, family 76, subfamily C, polypeptide 7), *AT5G01670* (NAD(P)-linked oxidoreductase superfamily protein), *AT5G04010* (F-box family protein), *AT5G22470* (poly ADP-ribose polymerase 3), *AT5G44310* (LEA protein), *AT5G45690* (histone acetyltransferase, DUF1264), *DOG1* (delay of germination 1), and *HVA22B* (HVA22 homolog B) begin their expression in mid seed development stages (embryos at late torpedo to early walking-stick stage). Only *AT4G36700* (RmlC-like cupins superfamily protein) has an initial expression in a very early developmental stage (heart embryo stage) [79].

#### 3.2.5. Cyan Module

The cyan module contains 1079 genes which are overexpressed in “Leaf”, “Protoplast”, “Root”, and “Trichome” tissues and underexpressed in “Flower”, “Seedling”, “Seed”, “Aerial tissue”, “Shoot apex”, “Inflorescence”, “Flower bud”, “Pollen”, “Petiole”, and “Developing leaf insertions” tissues. The enriched biological terms of this module are related to the defense response (Table 6).

*AT5G18490* (vacuolar sorting-associated protein DUF946) is the hub gene of this module. Its methylation is increased in multi-mutant drm1/drm2/cmt3 plants [80]. *ERF4* (ethylene responsive element binding factor 4), *ERF11* (ERF domain protein 11), *ERF5* (ethylene responsive element binding factor 5), *CEJ1* (cooperatively regulated by ethylene and jasmonate 1), *ERF6* (ethylene responsive element binding factor 6), *AT5G51190* (Integrase-type DNA-binding superfamily protein), *ACS6* (1-aminocyclopropane-1-carboxylic acid synthase 6), *MAPKKK14* (mitogen-activated protein kinase kinase kinase 14), and *MKK9* (MAP kinase kinase 9) control ethylene accumulation [81]. *SIP4* (SOS3-interacting protein 4), *STZ* (salt tolerance zinc finger), and *ZF2* (zinc-finger protein 2) are involved in salinity tolerance [81]. *VPS28-1* (vacuolar protein sorting-associated protein 28 homolog 1), *SRC2* (soybean gene regulated by cold-2), *ELC* (Ubiquitin-conjugating enzyme/RWD-like protein), *VPS2.1* (SNF7 family protein), and *VPS46.2* (SNF7 family protein) orchestrate trafficking from endosomes to central vacuole [81]. *AT1G02660* (α/β-Hydrolases superfamily protein), *IP5PII* (myo-inositol polyphosphate 5-phosphatase 2), *BAP1* (BON association protein 1), and *FAB1D* (FORMS APLOID AND BINUCLEATE CELLS 1A) participate in phospholipid signaling [81]. *SRO5* (similar to RCD one 5) controls reactive oxygen species (ROS) in plants, while *RHL41* (RESPONSIVE TO HIGH LIGHT 41) participates in signal transduction of ROS [81]. *NHL3* (NDR1/HIN1-like 3) and *PUB17* (plant U-box 17) function against *Pseudomonas syringae* and along with *AT2G34930* (disease resistance family protein/LRR family protein) are involved in biotic and abiotic stress conditions [81].

*CML38* (calmodulin-like 38), *AT3G10300* (Calcium-binding EF-hand family protein), *AT5G62570* (Calmodulin-binding protein-like protein), *CPK28* (calcium-dependent protein kinase 28), *CPK32* (calcium-dependent protein kinase 32), *AT4G34150* (Calcium-dependent lipid-binding domain family protein), and *AT4G27280* (Calcium-binding EF-hand family protein) are calcium-dependent genes [81]. *RPK1* (receptor-like protein kinase 1) and *CYP707A3* (cytochrome P450, family 707, subfamily A, polypeptide 3) are ABA-related genes [81]. Although the aforementioned genes are involved in specific defense responses, they all exhibit upregulation at any kind of environmental or biotic stress [81].

In addition, *CAF1b* (CCR4-associated factor 1b), *AT5G54940* (Translation initiation factor SUI1 family protein), *TEM1* (TEMPRANILLO 1), *SCL13* (SCARECROW-like 13), *MYBR1* (myb domain protein r1), *MYB73* (myb domain protein 73), *NAC102* (NAC domain-containing protein 102), *NAC062* (NAC domain-containing protein 62), *TIP* (TCV-interacting protein), *WRKY40* (WRKY DNA-binding protein 40), *WRKY33* (WRKY DNA-binding protein 33), *WRKY25* (WRKY DNA-binding protein 25), *WRKY11* (WRKY DNA-binding protein 11), *WRKY18* (WRKY DNA-binding protein 18), *HSFB2A* (heat shock transcription factor B2A), and *HSFA4A* (heat shock transcription factor A4A) are transcription factors upregulated in any stress condition [81].

*DIC2* (dicarboxylate carrier 2), *AT5G11650* (α/β-Hydrolases superfamily protein), *BCS1* (cytochrome BC1 synthesis), *AT2G46620* (P-loop containing nucleoside triphosphate hydrolases superfamily protein), *AT4G33920* (Protein phosphatase 2C family protein), *FC1* (ferrochelatase 1), *UCP5* (uncoupling protein 5), and *PNC2* (peroxisomal adenine nucleotide carrier 2) are involved in mitochondrial functions and are upregulated in any stress condition [81]. *RSH2* (RELA/SPOT homolog 2) encodes a homolog protein of RelA/SpoT, bacterial enzymes that adapt bacteria to various environmental stresses [81]. Finally, 51 transcription factors and 15 ubiquitin-ligase genes that are responsive to chitooctaose treatment [82] belong to this module.

#### 3.2.6. Blue Module

The blue module contains 3249 genes (22 of them are chloroplast ones), most of which are located in the same clade. They are overexpressed in “Leaf”, “Aerial tissue”, “Rosette”, “Seedling”, “Rosette leaf”, “Cotyledon”, and “Shoot” tissues and underexpressed in “Root”, “Cell culture”, “Root tip”, “Seed”, “Pollen”, “Flower”, “Lateral root”, “Pollen tube”, and “Phloem” tissues. The “photosynthesis”, “plastid organization”, “thylakoid membrane organization”, and “photosystem II assembly” GO terms and “chlorophyll A-B binding protein” and “PsbP” Pfam families of proteins are overrepresented (Table 7). The “chloroplast” Gene Ontology Cellular Component (GOCC) term is significantly overrepresented, indicating the localization of the proteins coded by the genes of this module. The proteome of chloroplasts is encoded mainly by the nuclear genome, although they require their own genome [83], supporting the grouping of a small number of chloroplast genes with numerous nuclear ones which are nevertheless enriched for chloroplast-specific biological terms.

*AT1G76450* (Photosystem II reaction center PsbP family protein) is the hub gene of this module. *AT1G76450*, *PSBP-1* (photosystem II subunit P-1), *PSBP-2* (photosystem II subunit P-2), *PPL1* (PsbP-like protein 1), *PnsL1* (Photosynthetic NDH subcomplex L 1), *PPD1* (PsbP-Domain Protein1), *AT2G28605* (Photosystem II reaction center PsbP family protein), *AT1G77090* (Mog1/PsbP/DUF1795-like photosystem II reaction center PsbP family protein), *PPD5* (PsbP domain protein 5), and *PPD6* (PsbP-domain protein 6) constitute the whole PsbP protein family. PsbP is a subcomplex of photosystem II which catalyses water splitting [72]. *CBL2* (calcineurin B-like protein 2) and *CBL10* (calcineurin B-like protein 10) encode calcium-signal sensors, and *CIPK1* (CBL-interacting protein kinase 1), *CIPK3* (CBL-interacting protein kinase 3), *CIPK7* (CBL-interacting protein kinase 7), *CIPK9* (CBL-interacting protein kinase 9), and *CIPK20* (CBL-interacting protein kinase 20), which encode proteins capable of interacting with CBLs, belong to this module [84]. *TOC33* (translocon at the outer envelope membrane of chloroplasts 33) regulates the expression of many genes that are involved in photosynthesis [85]. Tandem repeat genes *COR15A* (cold-regulated 15a) and *COR15B* (cold-regulated 15b), which are located in neighboring clades on the coexpression tree, have positively correlated expression [78].

### 3.3. Benchmarking of ACT2.6 Versus ACT2.0

For the coexpression clade produced in each of the 10 gene use cases tested, the top enriched GO:BP term of ACT2.0 and its corresponding adjP were recorded and compared to the adjP of the same term in the coexpression clade of ACT2.6 (Table 8).

In the case of *AT4G13170*, *HSP101*, *CTL2*, *AMS*, and *LHY*, the same biological terms were enriched, having very close FDR-adjusted *p*-values (adjP). Any small differences in adjP could be due to the different number of coexpressed genes that resulted in each webtool, rather than the difference in performance.

The gene coexpression subclades of multiple gene cases in ACT2.6 exhibited a “ladderization” effect, which is a characteristic of an unbalanced hierarchical tree. As a result, in the case of *COR15A*, *PSB28*, and *PSBT*, even when the internal node was set to 1, the resulting coexpression clade contained a large number of genes (far greater than the initial default number of 25). Consequently, the internal nodes in ACT2.0 were increased, as an attempt to match the number of genes of the clades of ACT2.6, as much as possible. In the case of *COR15A*, there were a lot of overlapping genes between the outcomes of ACT2.6 and ACT2.0, with the ACT2.0 coexpression clade exceeding the size of the ACT2.6 one. Nevertheless, the number of genes characterized by the most prominent GOBP term (“photosynthesis”) was similar between the two clades, resulting in ACT2.6 exhibiting smaller enrichment *p*-values than ACT2.0 for that term. In the case of *PSB28*, the expanded ACT2.0 clade of 262 genes exhibited almost identical adjP in the common top enriched “photosynthesis” GOBP term, with the ACT2.6 coexpression clade. In the case of *PSBT*, a comparable number of coexpressed genes between the tool versions could not be achieved. Thus, the smallest coexpression clade of *PSBT* that could be generated in ACT2.6, containing 995 coexpressed genes, was compared to the ACT2.0 coexpression clade of 4 internal nodes and 72 coexpressed genes which was originally tested as a use case. The resulting top enriched GOBP term was the same in each case (“photosynthesis”) with ACT2.6 having much lower adjP due to the ~14x higher gene number. Nevertheless, the coexpression clade of ACT2.0 contained all 72 chloroplast genes, while in the ACT2.6 coexpression clade of 995 genes, only 12 genes were chloroplast ones.

Finally, in the cases of *CEV1* and *emb1692*, ACT2.0 exhibited lower *p*-values for the same overrepresented biological terms. Specifically, the *CEV1* coexpression clade in ACT2.0 contained genes that were more relevant to the gene’s main function of primary cell wall formation [86]. On the other hand, the enriched terms of the *emb1692* clade were similar for both versions, with the adjP difference also being explained by the larger number of ACT2.0 coexpressed genes.

## 4. Discussion

### 4.1. Life Cycle of A. thaliana Through Gene Coexpression Patterns

#### 4.1.1. Seedling

The darkmagenta, blue, darkgreen, yellowgreen, darkolivegreen, and purple modules are overexpressed in “seedling” tissue in descending order of correlation. Out of these modules, the blue, yellowgreen, and darkmagenta modules are overexpressed in “aerial tissue”, while darkgreen, darkolivegreen, and purple exhibit their highest expression in “root” tissue. All aforementioned modules serve distinctly different biological purposes.

#### 4.1.2. Underground Development

Darkgreen and saddlebrown are the most overexpressed modules in the “root” tissue, followed by royalblue, steelblue, darkolivegreen, and purple, in decreasing order. Darkgreen is the only module that shows significant overexpression in all three “root”, “lateral root”, and “root tip” tissues. Consequently, there is a significant overrepresentation of “root morphogenesis” and “root development” GOBP terms within this module. Saddlebrown is overexpressed in “root” and “lateral root”, with ~86% of its genes being characterized by the “root system” POPA term. The darkolivegreen, royalblue, and purple modules showcase their highest overexpression in “root” and “cell culture” tissues. Those three modules contain mitochondrial complex 1 subunit genes [87], and therefore, the “mitochondrial protein complex” GOCC term is overrepresented in all three modules. The royalblue and darkolivegreen modules are unique in sharing LSM-family genes [68], with *LSM3B* participating as a hub gene in the royalblue module. The darkolivegreen module contains *AT3G60770* (Ribosomal protein S13/S15) as a hub gene which, as a ribosomal protein, is associated with the module’s overrepresented “translation” GOBP term. In addition, the royalblue and darkolivegreen modules share “Ribosome” as an overrepresented KEGG term, while purple shows overrepresentation of “Proteasome” KEGG term, supporting the identification as a hub gene of *AT4G24820* which encodes a 26S proteasome regulatory subunit. This observation is additionally supported by the non-cell-autonomous nature of lateral root development, wherein various cell types assume distinct functions and trigger a multitude of genetic networks throughout this progression [88].

The genes of black and magenta modules are overexpressed in “root” tissue along with “meristem”, “apex”, and “shoot apex”. Genes involved in the cell cycle are essential to root development [88], and these three modules share “cell cycle” as an overrepresented term, coupled with other similar terms. *HDA3* (histone deacetylase 3), *HD2B* (histone deacetylase 2B), and *HDT4* (histone deacetylase-related/HD-like protein), which belong to the black module, rearrange the structure of chromatin, affecting root development [88].

*ARF19* (auxin response factor 19) and *NPH4* (NON-PHOTOTROPHIC HYPOCOTYL) regulate *LBD16* (lateral organ boundaries-domain 16) and *LBD29* (lateral organ boundaries-domain 29) transcription factors which are responsible for lateral root initiation by promoting cell division. *ATARCA* (Transducin/WD40 repeat-like superfamily protein), *RACK1B_AT* (receptor for activated C kinase 1B), and *RACK1C_AT* (receptor for activated C kinase 1C), which are involved in cell cycle functions and are involved in lateral root development, are also regulated by *ARF19* and *NPH4* [88]. *NPH4*, *RACK1B_AT*, and *RACK1C_AT* belong to the black module, *ATARCA* belongs to the darkolivegreen module, and *ARF19* and *LBD16* belong to the darkgreen module.

In the early stages of lateral root development, where asymmetric cell division takes place, magenta module genes *CYCB;1* (CYCLIN B1;1), *BRXL4* (BREVIS RADIX-like 4), and *ATBRXL2* (Disease resistance/zinc finger/chromosome condensation-like region domain-containing protein) are induced. Also, magenta module genes *MP* (Transcriptional factor B3 family protein/auxin-responsive factor AUX/IAA-like protein) and *TMO6* (TARGET OF MONOPTEROS 6), which are related to each other as gene regulator and target gene, respectively, are expressed in the lateral root, affecting its development, although they are also involved in embryonic development [88]. In the ACT2.6 coexpression tree, *TMO6* and *BRXL4* are located in adjacent leaves, owing to their regulatory connection. *AT2G17500* (Auxin efflux carrier family protein) and *WRKY75* (WRKY DNA-binding protein 75), which belong to the darkgreen and cyan modules, respectively, both independently influence the structure of the root. Cyan module exhibits overexpression in “root” and “protoplast” tissues and is associated with the GOBP term “stress response”, justifying the place of *WRKY75* in this module, as it is mainly induced during phosphate starvation in roots [88].

#### 4.1.3. Aerial Development of the Plant

The blue, yellowgreen, and darkmagenta modules are overexpressed in “Aerial tissue”. The shoot apex is the tissue from which the total of the above-ground plant originates, except for hypocotyl and cotyledons. The shoot apex includes distinct layers of cells serving different biological functions [9]. Cells responsible for establishment and maintenance of the shoot apex show overexpression of *KNAT1* (homeobox knotted-like protein) and *KNAT6* (homeobox protein knotted-1-like 6), which belong in magenta module and are also adjacent genes in the ACT2.6 coexpression tree, *KNAT2* (homeobox knotted-like protein), which belongs in the turquoise module, and *STM* (SHOOT MERISTEMLESS) of the lightgreen module [9]. High-resolution single-cell RNA sequencing of shoot apex tissue exhibited a cluster of epidermal cell samples having a high accumulation of *HIS4* (histone 4) and *TSO2* (Ferritin/ribonucleotide reductase-like family protein) transcripts [9], which belong to the darkolivegreen module. Other epidermal cells of the shoot apex overexpress genes such as *CDKB2;1* (cyclin-dependent kinase B2;1), *CYCA1;1* (Cyclin A1;1), *ENODL15* (early nodulin-like protein 15), *MAD2* (MITOTIC ARREST-DEFICIENT 2), and *PCNA2* (proliferating cell nuclear antigen 2) of the magenta module, *CYCD3;2* (CYCLIN D3;2) and *PDF1* (protodermal factor 1) of the darkmagenta module, *RPL24* (plastid ribosomal protein L24), *ATML1* (MERISTEM LAYER 1), and *FDH* (formate dehydrogenase) of the blue module, and *RPS6* (RESISTANT TO P. SYRINGAE 6) of the turquoise module [9]. Finally, transcripts of *HIS4*, *CDKB2;1*, and *CYCA1;1* have also been found in proliferating cells [9].

#### 4.1.4. Photosynthetic Tissues

Blue and darkred are the only overexpressed modules in “rosette” tissue, where turquoise and paleturquoise are underexpressed, although their overexpression peaks in “rosette leaf” tissue. The blue, darkred, and yellowgreen modules are overexpressed in both “leaf” and “rosette leaf” tissues. The yellowgreen module contains chloroplast genes, exclusively, while the majority of blue module genes originate from the nuclear genome. They both share the “photosynthesis” GOBP term as the most significantly overrepresented one. Only the blue module is enriched with “chloroplast organization” GOBP term, confirming that although chloroplasts contain their own genome, most genes that are expressed in chloroplasts are encoded by the nuclear genome [83]. The darkred module is enriched with “defense response”, “response to other organism” and “response to stress” GOBP terms; as its hub genes, *SIB1* and *MEK1*, participate in the aforementioned processes. The darkred module also includes genes responsible for induced systemic resistance [89]. In addition, the cyan module includes many genes that are upregulated during various stresses [81], and it is enriched for all aforementioned darkred module enriched GOBP terms, as well as “response to chitin”. The Cyan module attains the top of its expression in “leaf” tissue, the same as the darkred module, but it is also overexpressed in “root” tissue, where the darkred module is significantly underexpressed.

Stomata, the structures that permit carbon dioxide uptake through leaves, indicate high expression of *SPCH* (SPEECHLESS) and *TMM* (TOO MANY MOUTHS) of the blue module, *AT4G31805* (WRKY family transcription factor) of the magenta module, and *BASL* of the darkmagenta module, in their early development [9].

#### 4.1.5. Phloem and Stem Tissue Development

Only the white module is significantly overexpressed in “basal tissue”, and its expression level peaks in “stem” tissue, justifying the enrichment of “plant-type secondary cell wall biogenesis” and “xylan metabolic process” GOBP terms, where *IRX3* and *GAUT12* hub genes participate, respectively. The steelblue module is also overexpressed in “stem” tissue, as it includes *NAC045* and *NAC086* which are linked to the module’s overrepresented “sieve element enucleation” and “sieve element differentiation” GOBP terms. Within vascular bundles in the stem, xylem and phloem tissues consist of specialized cell types including xylem fibers, xylem vessel elements, phloem sieve elements, and phloem companion cells, facilitating the transport of water and nutrients. In contrast to these highly specialized cells, cambium stem cells retain the ability to produce secondary xylem and phloem cells, augmenting the transport capacity and structural support of the growing shoot system [90]. *SEOR1* and *SEOa* of the steelblue module and *XCP1* (xylem cysteine peptidase 1) and *XCP2* (xylem cysteine peptidase 2) of the white module are expressed specifically in vascular tissues [9]. For xylem identification, the expression of *PXY* (Leucine-rich repeat protein kinase family protein) and *BHLH32* (basic helix–loop–helix 32), which belong to the magenta and darkgreen modules, respectively, is preceded by *XCP2* expression [9]. In addition, genes of the magenta and black modules, related to the auxin pathway, such as *MP* (MONOPTEROS), *LAX2* (like AUXIN RESISTANT 2), *PIN6* (Auxin efflux carrier family protein), and *IAA12* (AUX/IAA transcriptional regulator family protein), are overexpressed in xylem [9]. On the other hand, *AT5G57130* (Clp amino terminal domain-containing protein), *APL* (Homeodomain-like superfamily protein), *HAC2* (histone acetyltransferase of the CBP family 2), and *SEOR1*, which belong to the magenta, blue, turquoise, and steelblue modules, respectively, are mainly expressed in phloem [9].

The primary inflorescence stem comprises a wide range of tissues, spanning from the undifferentiated cambium stem cells to the terminally differentiated cells within the vasculature [90]. *ANT* (AINTEGUMENTA) of the magenta module exhibits expression in the cambium of the inflorescence stem, although it is also found to be expressed in root cambium cells. Especially in the cambium of the stem, *ANT* is coexpressed with *AT3G13980* (SKI/DACH domain protein) and *AT1G56210* (Heavy metal transport/detoxification superfamily protein), which belong to blue and magenta modules, respectively [90].

#### 4.1.6. Initiation and Development of Flower Tissue

The initial stages of flower growth are marked by significant alterations in shape and involve numerous transcriptional regulators overseeing crucial functions, such as establishing floral patterns and specifying floral organs [91]. In the early stages of flower primordium emergence, increased expression of *AP1*, which belongs to the lightgreen module, represses *SVP* (SHORT VEGETATIVE PHASE) of the blue module [91]. Also, *AG* and *AP3* of the lightgreen module showcase a high level of expression, temporally from the early stages of flower development, maintaining this situation until the late stages [91]. Lightgreen module genes, such as *AMS* which affects pollen wall formation, *SPL*, and *EMS1* (Leucine-rich repeat transmembrane protein kinase), are upregulated in the mid-stage of flower development [91]. The lightgreen and lightsteelblue1 modules show the highest expression in the “flower bud” and “flower” tissues, respectively. The Lightgreen module contains the *EXL6* hub gene which participates in pollen coat synthesis [66], in accordance with the most overrepresented GOBP term of the lightgreen module being “pollen wall assembly”. Lighsteelblue1 hub gene *MYB21* plays a crucial role in stamen development as a jasmonate regulator [55], explaining the most enriched GOBP term in this module being “stamen filament development”. The pink module is notably overexpressed in “pollen” and “pollen tube” tissues, exhibiting an overrepresentation in “pollen tube growth” and “pollen tube development” GOBP terms. In addition, the turquoise and paleturquoise modules also exhibit overexpression in “pollen” and “pollen tube” tissues, although their overexpression peaks in “rosette leaf” tissue. The lightgreen and sienna3 modules contain MADs-box transcription factors *SEP3* and *STK*, respectively, which combine their action to enable the transcriptional regulation of ovule target genes [92]. Also, *AT4G15750* (Plant invertase/pectin methylesterase inhibitor superfamily protein), a sienna3 hub gene, and many genes of the turquoise module are involved in the late stages of embryo sac development [57].

#### 4.1.7. Flower to Seed

The sienna3 module is overexpressed in the “flower” tissue and specifically female organs, like “pistil”, “silique”, “suspensor”, “embryo”, and “seed” tissues. The orange module combines overexpression in tissues associated with male reproduction, like “microsporocyte”, “pollen”, “anther”, and “seed”, although it is underexpressed in “flower” tissue. The darkorange module is most overexpressed in “silique”, like the sienna3 module, and is, generally, upregulated in all other specific tissues which are contained in silique, like “seed”, “endosperm”, “replum”, “embryo”, and “suspensor”. Its hub gene, *KCS18*, is associated with the “lipid metabolic process” GOBP term, which is also a prevalent module term.

The suspensor is an essential structure for further seed development, while also linking the embryo and further seed with the whole plant. *FUS3* of darkorange module affects the development of suspensors [93]. In “flower” tissue, the overexpression of the pink, turquoise, and lightgreen modules suggests a regulatory relationship where *WRKY2* (WRKY DNA-binding protein 2) governs the expression of *WOX8* (WUSCHEL related homeobox 8) and *AT2G33880* (homeobox-3), affecting suspensor development [93]. The midnightblue and darkorange modules are overexpressed in “endosperm”, “silique”, “embryo”, and “seed” tissues, with the latter being the tissue in which the genes of the the midnightblue module are mostly overexpressed, justifying the significantly overrepresented “seed development” GOBP term.

### 4.2. Comparison of ACT2.6 and ACT2.0

The main rationale behind the usage of WGCNA in the new ACT version (ACT2.6) was to discover whether the signed weighted TOM-based coexpression tree produced by WGCNA would outperform the coexpression results of the previous PCC-based ACT2.0 version (thus, the use of the same datasets and gene-describing biological terms was compulsory). In general, TOM provides a robust and accurate centrality measure that outperforms standard metrics in predicting gene importance, emphasizing the stronger correlation of intramodular connectivity with gene significance. Its consistent performance across various network contexts, especially when combined with soft thresholding techniques, ensures reliable preservation of biological signals and meaningful insights into gene interactions [4].

A main addition of ACT2.6 to ACT2.0 is the grouping of genes into modules, as well as the modules’ association with tissue traits. In general, the tissues in which each module is overexpressed align with the top enriched POPA terms for that module and are biologically consistent with the top enriched GO terms associated with the module’s genes (i.e., the yellowgreen module, being overexpressed in leaves, exhibits leaf-related POPA and photosynthesis-related GOBP enriched terms). Additionally, the top enriched GOBP term and the top overexpressed tissue of each module coincided with the biological terms of the module’s hub gene, as well as its tissue-specific overexpression, in over half of the modules (Table 1). Since the best way to evaluate a coexpression tool is to study whether it is able to replicate known biology, these concordances served as positive controls for the ability of ACT2.6 to produce functionally relevant coexpression partners.

Furthermore, the identification of modules of coexpressed genes allowed for a better evaluation of the coexpression clades produced by ACT2.6, compared to those of the previous ACT version. For example, the lightsteelblue1 module links *MYB21* and *MYB24*, which encode jasmonate regulators responsible for stamen and pollen development [55], with *AT5G44630* and other terpene synthases which may be involved in pollinator attraction or the protection of reproductive organs against bacteria and fungi [73]. In contrast, these two genes are distant in the ACT2.0 tree. In ACT2.6, *SUS2* and *LEC1*, which affects the former’s expression [94], belong to the same subtree of 81 genes that participate exclusively in the darkorange module, while in ACT2.0, those two genes belong in distinctly different clades. *AT1G76640* (Calcium-binding EF-hand family protein) and *AGD11* (ARF-GAP domain 11) are calmodulin-like proteins that are upregulated during pollen germination and pollen tube growth [95]. In ACT2.0, *AT1G76640* and *AGD11* belong to separate subtrees, while in ACT2.6, they both belong to a clade of 631 exclusively pink module genes. Finally, *COW1* and *RHD2* participate in hair root development [96] and are located in the same subtree of 159 genes of the darkgreen module in ACT2.6, while in ACT2.0, *COW1* and *RHD2* belong to separate clades.

In summary, ACT2.6 WGCNA-derived coexpression clades were more unbalanced compared to ACT2.0 ones. Nevertheless, the benchmarking for the creation of coexpression clades of the 10 use-case genes showed that ACT2.6 and ACT2.0 produced, in general, comparable results, regarding the pathway participation of genes and the discovery of potential functional partners. However, there were exceptions where one tool outperformed the other. Therefore, it is recommended that users consult both ACT2.6 and ACT2.0 when they focus on the topology of the coexpression clades.

ACT2.6 constitutes a major upgrade over ACT2.0 by producing coexpressed gene modules, identifying intramodular hub genes, and introducing module–tissue trait associations, all of which are features unique to this version. Additionally, ACT2.6 identified multiple gene partners that were not discovered by ACT2.0, located within the same module and coexpression clade. Therefore, ACT2.6 is a significant addition in the field of gene coexpression tools when discovering gene partners for a gene of interest or attributing biological roles to genes of unknown function.

### 4.3. Limitations

The limitation of the depiction of the coexpression tree produced by hierarchical clustering, as in ACT2.6, is the inability to portray negative gene correlations. In addition, through this approach, genes may only participate in a single coexpression clade, a limitation that is also present in the WGCNA module discovery analysis, as genes are grouped into non-overlapping modules. This is contrary to the fact that a gene may interact with different sets of genes, playing diverse biological roles, while also impeding the identification of inter-modular genes, i.e., genes that act as links between distinct functional modules.

Microarrays are constrained by the presence of probes for studying genes and potential distortions due to cross-hybridization, particularly when using the default CDF. These limitations are addressed by RNA-Seq, which is progressively replacing microarrays, as the amount of publicly available RNA-Seq data for *A. thaliana* now surpasses that of microarray data. While ACT2.6 does not include RNA-Seq data in its gene expression analysis, limiting the number of genes that can be studied, it uses the constantly updated Brainarray CDF, which incorporates the latest knowledge of the human genome and transcriptome and ensures that each probe set corresponds to one gene, and vice versa. In addition, even though RNA-Seq offers greater sensitivity, gene expression values between microarrays and RNA-Seq are largely comparable, especially in genes of average expression levels [97], while GCNs produced by microarrays and RNA-Seq produce similar coexpression values and enrichments [98,99]. Finally, RNA-Seq has not yet fully replaced microarrays, as the optimal normalization method for RNA-Seq-based gene coexpression analysis remains under debate, whereas microarray normalization algorithms have been extensively refined over time.

## Figures and Tables

**Table 1:** The 27 merged functional modules determined by WGCNA. The top enriched GOBP term of each module and its corresponding FDR-adjusted *p*-value using ACT2.6 built-in enrichment analysis are displayed in each case. Underlined modules are presented in the Results section. The top overexpressed tissues and hub genes of each module are displayed. The last two columns signify whether the hub gene is described by its module’s top enriched term and whether the overexpression of the hub gene is bibliographically supported in the module’s top overexpressed tissue.

Module	Number of Genes	Top Enriched GO Biological Process Term	Adj. *p*-Value	Top Overexpressed Tissue	Top Hub Gene	Concordance of Hub Gene and Top Enriched BP Term	Evidence of Hub Gene Overexpression in Top Tissue
lightsteelblue1	27	stamen filament development	4.0 × 10^−5^	Flower	MYB21	Yes	[55]
plum1	39	N/A	N/A	Rosette Leaf	AT5G41250	N/A	N/A
yellowgreen	45	photosynthesis	6.6 × 10^−21^	Rosette Leaf	ATPH	No	[56]
sienna3	47	protein depalmitoylation/negative regulation of molecular function	4.4 × 10^−3^	Silique	AT4G15750	Yes	[57]
darkmagenta	48	external encapsulating structure organization/cell wall organization	2.2 × 10^−2^	Flower Bud/Shoot Apex	SBT1.3/HTH	No	[58,59]
violet	57	response to heat	2.5 × 10^−42^	Seed	HSP101	Yes	[60]
paleturquoise	67	N/A	N/A	Rosette Leaf	CID13	N/A	No
steelblue	87	callose deposition in phloem sieve plate	4.4 × 10^−6^	Root	MLO13	No	[61]
saddlebrown	92	cell surface receptor signaling pathway	6.1 × 10^−3^	Root	AT5G59930	Yes	[62]
white	102	plant-type secondary cell wall biogenesis	8.4 × 10^−38^	Stem	IRX3	Yes	[63]
darkorange	107	secondary metabolic process	1.5 × 10^−2^	Silique	KCS18	Yes	[64]
orange	116	N/A	N/A	Microsporocyte	AT5G20370	N/A	N/A
darkred	167	defense response	7.2 × 10^−33^	Leaf	SIB1	Yes	[65]
lightgreen	329	pollen wall assembly/cellular component assembly involved in morphogenesis/extracellular matrix assembly	4.6 × 10^−15^	Flower Bud	EXL6	Yes	[66]
lightcyan	356	DNA metabolic process	1.1 × 10^−12^	Flower	AT5G02520	N/A	No
midnightblue	385	seed development	2.3 × 10^−17^	Seed	AT1G72100	Yes	[67]
royalblue	401	autophagy/protein localization	9.3 × 10^−6^	Root	LSM3B	No	[68]
greenyellow	702	signaling/signal transduction	3.5 × 10^−2^	Leaf	AT1G32040	N/A	N/A
magenta	832	cell cycle	8.1 × 10^−59^	Root	NP3	No	[69]
darkolivegreen	836	translational elongation	1.0 × 10^−112^	Root	AT3G60770	Yes	No
pink	836	pollen tube growth	1.1 × 10^−29^	Pollen	CPK24	Yes	[12,70]
cyan	1079	response to chitin	5.7 × 10^−54^	Leaf	AT5G18490	N/A	[71]
darkgreen	1930	secondary metabolic process	4.8 × 10^−14^	Root	AT4G28890	Yes	No
purple	1933	vesicle-mediated transport	5.3 × 10^−29^	Root	AT4G24820	No	No
blue	3249	photosynthesis	6.2 × 10^−86^	Leaf	AT1G76450	Yes	[72]
turquoise	3593	regulation of transcription, DNA-templated/regulation of RNA biosynthetic process	4.9 × 10^−15^	Rosette Leaf	AT3G58390	Yes	No
black	3809	RNA processing	1.7 × 10^−70^	Seed	MIRO1	No	No

N/A: not applicable. Underlines denote the specific modules that are presented in the following sections in results.

**Table 2:** Lightsteelblue1 module enriched biological terms.

Category	*p*-Value	Term ID	Description
Gene Ontology: Biological Process	4.0 × 10^−5^	GO:0080086	stamen filament development
	1.3 × 10^−4^	GO:0046246	terpene biosynthetic process
	2.7 × 10^−4^	GO:0042214	terpene metabolic process
	3.8 × 10^−4^	GO:0071836	nectar secretion
Gene Ontology: Molecular Function	1.6 × 10^−6^	GO:0010333	terpene synthase activity
	1.6 × 10^−6^	GO:0016838	carbon–oxygen lyase activity, acting on phosphates
	1.2 × 10^−4^	GO:0050551	myrcene synthase activity
Plant Ontology: Plant Anatomy	3.3 × 10^−8^	PO:0009056	flower nectary
	3.3 × 10^−8^	PO:0009035	Nectary
	1.0 × 10^−7^	PO:0005656	portion of secretory tissue
KEGG	9.8 × 10^−8^	ath00902	monoterpenoid biosynthesis
AraCyc	2.3 × 10^−5^	PWY-3041	monoterpene biosynthesis
Pfam	1.3 × 10^−7^	Terpene_synth_C	terpene synthase family, metal binding domain
	1.3 × 10^−7^	Terpene_synth	terpene synthase, N-terminal domain

**Table 3:** Yellowgreen module enriched biological terms.

Category	*p*-Value	Term ID	Description
Gene Ontology: Biological Process	6.6 × 10^−21^	GO:0015979	photosynthesis
	4.3 × 10^−17^	GO:0019684	photosynthesis, light reaction
	4.0 × 10^−14^	GO:0006091	generation of precursor metabolites and energy
	9.0 × 10^−12^	GO:0022900	electron transport chain
Gene Ontology: Molecular Function	2.3 × 10^−8^	GO:0048038	quinone binding
	4.5 × 10^−8^	GO:0008137	NADH dehydrogenase (ubiquinone) activity
	4.5 × 10^−8^	GO:0003735	structural constituent of ribosome
	6.4 × 10^−8^	GO:0045156	electron transporter, transferring electrons within the cyclic electron transport pathway of photosynthesis activity
Gene Ontology: Cellular Component	1.0 × 10^−37^	GO:0044435	plastid part
	8.1 × 10^−33^	GO:0009507	chloroplast
	5.3 × 10^−31^	GO:0009534	chloroplast thylakoid
	7.0 × 10^−29^	GO:0009535	chloroplast thylakoid membrane
Plant Ontology: Plant Anatomy	3.7 × 10^−8^	PO:0020030	cotyledon
	3.7 × 10^−8^	PO:0025099	embryo plant structure
	3.7 × 10^−8^	PO:0025233	portion of embryo plant tissue
Plant Ontology: Plant Structure Development Stage	1.6 × 10^−7^	PO:0007095	LP.08 eight leaves visible stage
	3.1 × 10^−7^	PO:0001050	leaf development stage
AraCyc	1.3 × 10^−8^	PWY-101	photosynthesis light reactions
Pfam	1.3 × 10^−5^	Photo_RC	photosynthetic reaction center protein
	1.3 × 10^−5^	PSII	photosystem II protein

**Table 4:** White module enriched biological terms.

Category	*p*-Value	Term ID	Description
Gene Ontology: Biological Process	8.4 × 10^−38^	GO:0009834	plant-type secondary cell wall biogenesis
	4.2 × 10^−34^	GO:0042546	cell wall biogenesis
	1.9 × 10^−32^	GO:0009832	plant-type cell wall biogenesis
	2.6 × 10^−19^	GO:0045491	xylan metabolic process
Gene Ontology: Molecular Function	5.1 × 10^−9^	GO:0052716	hydroquinone:oxygen oxidoreductase activity
	8.3 × 10^−8^	GO:0016682	oxidoreductase activity, acting on diphenols and related substances as donors, oxygen as acceptor
Gene Ontology: Cellular Component	8.9 × 10^−5^	GO:0000139	Golgi membrane
Plant Ontology: Plant Anatomy	7.0 × 10^−25^	PO:0005352	xylem
	2.0 × 10^−14^	PO:0005849	primary xylem
	2.3 × 10^−14^	PO:0005598	vascular cambium
	4.9 × 10^−13^	PO:0005848	secondary xylem
Plant Ontology: Plant Structure Development Stage	1.1 × 10^−8^	PO:0001083	inflorescence development stage
KEGG	2.6 × 10^−2^	ath00520	amino sugar and nucleotide sugar metabolism
AraCyc	2.9 × 10^−4^	PWY-1001	cellulose biosynthesis
AtRegNet	1.1 × 10^−17^	AT2G44730	alcohol dehydrogenase transcription factor Myb/SANT-like family protein
	5.3 × 10^−16^	AT1G61730	DNA-binding storekeeper protein-related transcriptional regulator
	9.2 × 10^−11^	AT2G21230	basic-leucine zipper (bZIP) transcription factor family protein
Plant Cistrome Database	2.0 × 10^−2^	AT5G47660	homeodomain-like superfamily protein
Pfam	4.6 × 10^−7^	Cu-oxidase_3	multicopper oxidase
	9.5 × 10^−6^	Glyco_hydro_10	glycosyl hydrolase family 10

**Table 5:** Midnightblue module enriched biological terms.

Category	*p*-Value	Term ID	Description
Gene Ontology: Biological Process	2.3 × 10^−17^	GO:0048316	seed development
	7.4 × 10^−12^	GO:0010431	seed maturation
	5.6 × 10^−7^	GO:0010344	seed oilbody biogenesis
Gene Ontology: Molecular Function	5.7 × 10^−10^	GO:0045735	nutrient reservoir activity
Gene Ontology: Cellular Component	3.2 × 10^−11^	GO:0005811	lipid droplet
Plant Ontology: Plant Anatomy	2.0 × 10^−2^	PO:0009089	endosperm
Plant Ontology: Plant Structure Development Stage	1.0 × 10^−23^	PO:0007632	seed maturation stage
KEGG	3.1 × 10^−4^	ath04075	plant hormone signal transduction
AraCyc	2.3 × 10^−2^	PWY-5060	luteolin biosynthesis
AtRegNet	3.9 × 10^−4^	AT5G23930	mitochondrial transcription termination factor family protein
Plant Cistrome Database	1.3 × 10^−4^	AT5G07310	integrase-type DNA-binding superfamily protein
Pfam	3.7 × 10^−8^	Oleosin	oleosin

**Table 6:** Cyan module enriched biological terms.

Category	*p*-Value	Term ID	Description
Gene Ontology: Biological Process	5.7 × 10^−54^	GO:0010200	response to chitin
	2.0 × 10^−53^	GO:0010243	response to organonitrogen compound
	2.0 × 10^−53^	GO:0006952	defense response
Gene Ontology: Molecular Function	4.0 × 10^−16^	GO:0004672	protein kinase activity
Gene Ontology: Cellular Component	9.1 × 10^−16^	GO:0005886	plasma membrane
	5.7 × 10^−14^	GO:0071944	cell periphery
	4.0 × 10^−11^	GO:0016020	membrane
	1.2 × 10^−4^	GO:0036452	ESCRT complex
Plant Ontology: Plant Anatomy	3.7 × 10^−62^	PO:0002000	stomatal complex
	3.7 × 10^−62^	PO:0000293	guard cell
	7.0 × 10^−62^	PO:0025165	shoot epidermal cell
Plant Ontology: Plant Structure Development Stage	1.8 × 10^−53^	PO:0007123	LP.06 six leaves visible stage
KEGG	1.5 × 10^−16^	ath04626	plant–pathogen interaction
AtRegNet	1.8 × 10^−47^	AT3G42860	zinc knuckle (CCHC-type) family protein
	1.5 × 10^−23^	AT4G16150	calmodulin-binding transcription activator 5
Plant Cistrome Database	1.3 × 10^−47^	AT3G42860	zinc knuckle (CCHC-type) family protein
Pfam	9.0 × 10^−15^	AP2	AP2 domain
	2.2 × 10^−13^	Pkinase_Tyr	protein tyrosine kinase

**Table 7:** Blue module enriched biological terms.

Category	*p*-Value	Term ID	Description
Gene Ontology: Biological Process	6.2 × 10^−86^	GO:0015979	photosynthesis
Gene Ontology: Molecular Function	4.8 × 10^−11^	GO:0016491	oxidoreductase activity
Gene Ontology: Cellular Component	0.0 × 10^+0^	GO:0009507	chloroplast
Plant Ontology: Plant Anatomy	0.0 × 10^+0^	PO:0000013	cauline leaf
Plant Ontology: Plant Structure Development Stage	0.0 × 10^+0^	PO:0001050	leaf development stage
WikiPathways	3.8 × 10^−4^	WP2622_r85067	starch metabolism
KEGG	1.5 × 10^−24^	ath00195	photosynthesis
AraCyc	1.5 × 10^−12^	PWY-101	photosynthesis light reactions
AtRegNet	1.1 × 10^−18^	AT1G71450	integrase-type DNA-binding superfamily protein
Plant Cistrome Database	2.2 × 10^−18^	AT1G71450	integrase-type DNA-binding superfamily protein
Pfam	1.2 × 10^−8^	Chloroa_b-bind	chlorophyll A-B binding protein

**Table 8:** Comparison of the coexpression results of the gene use cases of ACT2.0 versus the new 2.6 version of the webtool. The adjP of the top enriched Gene Ontology Biological Process term of ACT2.0 is compared with the corresponding adjP of this term in ACT2.6.

Input Gene	ACT2.0 Coexpressed Gene Number	ACT2.0 Tree Internal Nodes	ACT2.6 Coexpressed Gene Number	ACT2.6 Tree Internal Nodes	Top ACT2.0 Enriched GOBP Term	ACT2.0 Adj. *p*-Value	ACT2.6 Adj. *p*-Value	Common Coexpressed Genes
*AT4G13170*	135	6	160	1	translational elongation	1.3 × 10^−175^	1.0 × 10^−174^	130
*HSP101*	26	8	26	13	response to heat	1.5 × 10^−37^	2.3 × 10^−38^	23
*COR15A*	1834	12	1426	1	photosynthesis	6.4 × 10^−94^	1.1 × 10^−121^	1321
*CEV1*	31	8	66	4	polysaccharide biosynthetic process	1.2 × 10^−14^	4.7 × 10^−3^	10
*CTL2*	25	18	25	19	plant-type secondary cell wall biogenesis	5.5 × 10^−27^	6.4 × 10^−24^	20
*PSB28*	265	14	225	2	photosynthesis	1.0 × 10^−98^	7.9 × 10^−99^	207
*LHY*	24	9	14	8	rhythmic process	3.2 × 10^−11^	9.8 × 10^−8^	12
*PSBT*	72	4	995	1	photosynthesis	5.8 × 10^−44^	3.1 × 10^−134^	12
*AMS*	92	8	91	1	pollen exine formation	5.4 × 10^−18^	2.9 × 10^−17^	83
*emb1692*	68	8	42	17	embryo development	1.1 × 10^−4^	2.6 × 10^−2^	15

## Data Availability

The list of microarray samples used in this study is available in https://www.michalopoulos.net/act2.6/sample_table.php (accessed on 8 January 2025).
